# Development and Validation of a Combined Ferroptosis and Immune Prognostic Classifier for Hepatocellular Carcinoma

**DOI:** 10.3389/fcell.2020.596679

**Published:** 2020-12-23

**Authors:** Yang Liu, Xi Zhang, Junjun Zhang, Juan Tan, Jie Li, Zewen Song

**Affiliations:** ^1^Department of Pathology, The Third Xiangya Hospital of Central South University, Changsha, China; ^2^Department of Oncology, The Third Xiangya Hospital of Central South University, Changsha, China; ^3^Department of Information Science and Engineering, Hunan University of Chinese Medicine, Changsha, China

**Keywords:** ferroptosis, immune, hepatocellular carcinoma, prognosis, personalized therapy

## Abstract

**Background:**

Immunotherapy and sorafenib exert anti-tumor effects via ferroptosis, but reliable biomarkers for the individual treatment and prognosis prediction of hepatocellular carcinoma (HCC) based on the ferroptosis and immune status remain lacking.

**Methods:**

Ferroptosis-related genes (FRGs) were identified by downloading data from FerrDb and by searching and reading original articles from PubMed. Immune-related genes (IRGs) were downloaded from ImmPort. Prognostic FRGs and IRGs in the GSE14520 (*n* = 220) and The Cancer Genome Atlas (TCGA, *n* = 365) datasets were identified. Least absolute shrinkage and selection operator (LASSO) Cox regression and multivariate Cox regression were used for model construction. Ferroptosis expression profiles, the infiltration of immune cells, and the somatic mutation status were analyzed and compared.

**Results:**

Twenty-seven prognostic ferroptosis- and immune-related signatures were included to construct a comprehensive index of ferroptosis and immune status (CIFI). A subgroup of patients was identified as having a high CIFI value, which was associated with a worse prognosis. This subgroup of patients had significantly up-regulated expressions of many suppressors of ferroptosis and higher fractions of immunosuppressive cells, such as cancer-associated fibroblasts (CAFs) and myeloid-derived suppressor cells (MDSCs). Notably, somatic mutation analysis indicated that high-CIFI patients had higher levels of tumor heterogeneity and higher mutation frequencies of genes like TP53.

**Conclusion:**

In this work, a novel prognostic classifier was developed based on ferroptosis- and IRGs in HCC, and this classifier could be used for prognostic prediction and the selection of patients for immunotherapies and targeted therapies.

## Introduction

Hepatocellular carcinoma (HCC) is a lethal disease with high incidence and dismal prognosis (Hartke et al., 2017; Kulik and El-Serag, 2019). Even among patients who receive resection at an early stage, HCC reoccurs in more than half within 2 years (Tabrizian et al., 2015). Sorafenib and lenvatinib are the two first-line therapies for advanced HCC patients, who show a medium overall survival (OS) of around 1 year with the treatment (Llovet et al., 2008; Kudo et al., 2018). Many phase I and II clinical trials suggest that immune-based therapies, including anti-CTLA-4, anti-PD-1, and anti-PD-L1 strategies, benefit HCC patients (Hilmi et al., 2019). A recent phase III study also indicated that unresectable HCC patients who receive atezolizumab plus bevacizumab as a first-line treatment have a 1-year survival rate of 67.2% and a median progression-free survival of 6.8 months, which are better than those of patients who receive only sorafenib (Finn et al., 2020a). However, another immune checkpoint inhibitor, pembrolizumab, failed to reach its primary end-points as a second-line therapy against HCC in a randomized, double-blind, phase III trial (Finn et al., 2020b). Nivolumab also failed to prove its superiority over sorafenib in a phase III trial (Liu Z. et al., 2019). These results suggest that HCC might have a complex immune status, and more studies are required to understand its underlying mechanisms.

Ferroptosis might be one such essential mechanism in HCC that deserves attention. As an iron-dependent, lipid peroxidation-mediated form of cell death, ferroptosis is distinct from apoptosis, necrosis, and autophagy (Dixon et al., 2012). Since its first definition in 2012, numerous genes have been identified to regulate this new form of cell death (Dixon et al., 2012; Hassannia et al., 2019). Some are well-defined suppressors of ferroptosis (SOFs), some are drivers of ferroptosis (DOFs), and some genes, like TP53, could act as a SOF or DOF in a context-dependent manner (Jiang et al., 2015; Tarangelo et al., 2018). A remarkable study was conducted by Wang W. et al. (2019), who reported that CD8+ T cells enhance ferroptosis by down-regulating SLC3A2 and SLC7A11, and the induction of ferroptosis contributes to the anti-tumor efficacy of immunotherapy, suggesting that the immune system might, at least in part, function through ferroptosis (Stockwell and Jiang, 2019). Moreover, ferroptotic cancer cells might release signals like oxidized lipid mediators to affect anti-tumor immunity, or a small proportion of cells undergoing ferroptosis might suppress the immune system and allow tumor growth (Friedmann Angeli et al., 2019). However, a comprehensive regulatory network between ferroptosis and immune response is currently lacking, as few studies have explored their relationship.

Sorafenib, the standard first-line therapy against advanced HCC, also exerts cytotoxic effects via the induction of ferroptosis (Louandre et al., 2013, 2015; Galmiche et al., 2014). Several genes have been reported to regulate the sensitivity of HCC cells to sorafenib by enhancing or inhibiting ferroptosis (Louandre et al., 2015; Sun et al., 2016a; Feng et al., 2020). For instance, the down-regulation of acyl-CoA synthetase long-chain family member 4 (ACSL4) attenuates sorafenib-induced lipid peroxidation and ferroptosis (Feng et al., 2020). Additionally, the sorafenib-induced up-regulation of metallothionein 1G (MT1G) in HCC suppresses ferroptosis and contributes to the acquired sorafenib resistance of the disease (Sun et al., 2016a).

Consequently, ferroptosis, a biological process in which immune therapy and sorafenib converge to exert an anti-tumor effect, might have a fundamental impact on the treatment response of HCC. The understanding of the occurrence and regulation of ferroptosis has increased considerably in recent decades; however, only a few studies have explored the ferroptosis-related genes (FRGs) and pathways in HCC (Louandre et al., 2013, 2015; Carlson et al., 2016; Houessinon et al., 2016; Sun et al., 2016b; Bai et al., 2019; Qi et al., 2019; Feng et al., 2020; Kim et al., 2020). With the currently available FRGs and immune-related genes (IRGs), and the accumulative data deposited in public databases like The Cancer Genome Atlas (TCGA), it is hypothesized that a prognostic molecular classifier based on the immune response and ferroptosis status might help to identify subgroups of HCC patients with distinct ferroptosis-immune phenotypes and survival profiles. In the present work, it is demonstrated that a comprehensive index of ferroptosis and immune status (CIFI) developed from FRGs and IRGs is tightly correlated with the actual ferroptosis and immune status, as well as the prognosis of HCC patients.

## Materials and Methods

### Study Population and Data Acquisition

Publicly available data on HCC were downloaded from the Gene Expression Omnibus (GEO) database (GSE14520/GPL3921) and TCGA, and were processed as reported in the authors’ previous study (Zhang J. et al., 2020). Patients who met the following selection criteria were included: (a) histologically diagnosed with HCC; (b) available gene expression data; (c) available survival information. The first sample according to the label was selected if the same patient had two or more samples in the TCGA dataset. The baseline characteristics of patients in these two cohorts are summarized in Table 1.

**TABLE 1:** Baseline characteristics of HCC patients in the GSE14520 and TCGA cohorts.

Clinical characteristics	GSE14520		TCGA	
	Total (*n* = 220)	%	Total (*n* = 365)	%
**Age**				
<60	178	80.9	165	45.2
≥60	42	19.1	200	54.8
**Gender**				
Female	30	13.6	119	32.6
Male	190	86.4	246	67.4
**T**				
T1	–	–	180	49.3
T2	–	–	91	24.9
T3	–	–	78	21.4
T4	–	–	13	3.6
Unknown	–		3	0.8
**N**				
N0	–	–	248	67.9
N1	–	–	4	1.1
Nx	–	–	113	31.0
**M**				
M0	–	–	263	72.1
M1	–	–	3	0.8
Mx	–	–	99	27.1
**Stage**				
I	93	42.3	170	46.6
II	77	35	84	23.0
III	48	21.8	83	22.7
IV	0	0	4	1.1
Unknown	2	0.9	24	6.6
**Adjacent hepatic tissue inflammation**				
None	–	–	117	32.1
Mild	–	–	98	26.8
Severe	–	–	17	4.7
Unknown	–	–	133	36.4
**Vascular tumor cell type**				
None	–	–	205	56.2
Micro	–	–	91	24.9
Macro	–	–	16	4.4
Unknown	–	–	53	14.5
**Ishak fibrosis score**				
No fibrosis	–	–	74	20.3
Portal fibrosis	–	–	31	8.5
Fibrous septa	–	–	27	7.4
Nodular formation and incomplete cirrhosis	–	–	9	2.5
Established cirrhosis	–	–	68	18.6
Unknown	–	–	156	42.7
**AFP**				
≥300	118	53.6	66	18.1
<300	99	45	212	58.1
Unknown	3	1.4	87	23.8
**Child–Pugh**				
A	–	–	216	59.2
B	–	–	21	5.8
C	–	–	1	0
Unknown	–	–	127	34.8
**Status**				
Alive	136	61.8	234	64.4
Dead	84	38.2	130	35.6
Unknown	–	–	1	0
**HBV status**				
AVR-CC	56	25.5	–	–
CC	155	70.5	–	–
Unknown	9	4	–	–
**Main tumor size**				
Small	139	63.2	–	–
Large	80	36.4	–	–
Unknown	1	0	–	–
**Multi-nodular**				
No	175	79.5	–	–
Yes	45	20.5	–	–
**Cirrhosis**				
No	18	8.2	–	–
Yes	202	91.8	–	–
**BCLC**				
A	148	67.3	–	–
B	22	10	–	–
C	28	12.7	–	–
Unknown	22	10	–	–
**Histologic grade**				
G1			55	15.1
G2			175	47.9
G3			118	32.3
G4			12	3.3
Unknown			5	1.3
**CIFI**				
Low	140	63.6	232	63.6
High	80	36.4	133	36.4

### Generation of FRGs and IRGs

The FRGs were first downloaded from the FerrDb website.^1^ Because the database was last updated on February 23, 2020, PubMed^2^ was searched with the keyword “ferroptosis” on July 13, 2020, and 1,113 records were obtained. The authors read all the original articles and supplemented the list of FRGs (Supplementary Table 1) with the following criteria: (a) the inhibitor(s) or activator(s) of the gene could regulate ferroptosis; (b) the over-expression or down-regulation of the gene could regulate ferroptosis. Non-coding RNAs (ncRNAs) and markers of ferroptosis were not included. A total of 283 FRGs were identified, and are summarized in Supplementary Table 1. These FRGs were further divided into two groups based on their regulatory effects on ferroptosis, namely DOFs and SOFs.

The comprehensive list of IRGs was downloaded from ImmPort^3^ on August 2, 2020, as reported in other studies (Li et al., 2017; Zheng et al., 2020). The FRGs and IRGs available in the GSE14520 and TCGA datasets were included in this study.

### Development of the CIFI

The FRGs and IRGs in the GSE14520 and TCGA were first respectively subjected to univariate Cox regression. The genes with prognostic significance (*p* < 0.05) in both datasets were input into a least absolute shrinkage and selection operator (LASSO) Cox regression model, which is a widely used machine learning algorithm that deals with multicollinearity (Gui and Li, 2005; Wu et al., 2019). The analysis generated crucial genes for model construction, and was achieved by using the “glmnet” package in R software (Friedman et al., 2010). A multivariate Cox regression model was applied to obtain the regression coefficients for these crucial genes. To reflect the comprehensive effects of the ferroptosis and immune status, a risk score of each patient was calculated by multiplying the normalized gene expression of each crucial gene with its corresponding regression coefficient. To facilitate the interpretation of results from different datasets, the CIFI value of each patient was calculated by using the risk score of the patient subtracted by the minimum risk score of the cohort, which was then divided by the maximum risk score of the cohort, namely CIFI = (Risk score−Min)/Max.

### Functional Analysis and Heterogeneity

Gene Set Enrichment Analysis (GSEA software, version 4.0.3) was used to investigate the pathways enriched in the high- and low-CIFI subgroups. Gene expression data were loaded into GSEA, and *c2.cp.v7.1.symbols.gmt* was selected as the gene set database. The pathways with the following criteria were regarded to be significantly enriched: nominal *p*-value < 0.05, false discovery rate (FDR) *q*-value < 0.25, and normalized enrichment score (NES) > 1.

The crucial genes were constructed into a protein–protein interaction (PPI) network by uploading them into the STRING database.^4^ The network was visualized by Cytoscape (version 3.8.0). The expressions of these genes in tumor and normal samples were visualized by the “pheatmap” package in R. Principal component analysis (PCA) was carried out to examine the clustering efficacy of the prognostic signature with the “gmodels” package in R.

Somatic mutation data of the TCGA cohort were downloaded from the GDC database on August 7, 2020. The downloaded MAF files of simple nucleotide variation (workflow type: varScan2 variant aggregation and masking) were processed and visualized by the “maftools” package in R. The tumor mutation burden (TMB) and the mutant-allele tumor heterogeneity (MATH) score of tumor samples in the TCGA cohort were also calculated by the “maftools” package.

### Immune Profile Analysis

To analyze the immune status of each sample in the GSE14520 and TCGA cohorts, the relative infiltrations of 28 immune cell types in the tumor microenvironment (TME) were calculated via single-sample GSEA (ssGSEA) with the application of the “GSVA” package in R. The feature gene panels for each type of immune cell were downloaded from the publication by Charoentong et al. (2017). The normalized gene expression data of the GSE14520 and TCGA cohorts were further uploaded into the EPIC website,^5^ and the proportions of eight categories of immune cells were estimated according to the instructions on the website (Racle et al., 2017).

### Statistical Analysis

Univariate and multivariate Cox regressions were conducted by using the “survminer” package in R. The OS, relapse-free survival (RFS), disease-specific survival (DSS), and progression-free interval (PFI) of the high- and low-CIFI subgroups were compared using the Kaplan–Meier method with a log-rank test. Time-dependent receiver operating characteristic (ROC) analyses and the subsequent calculation of the area under the curve (AUC) were performed using the “timeROC” package in R. The “corrr” and “corrplot” packages were used to conduct the correlation analysis with the Pearson method. The significance of the differences in the expressions of specific genes or in the fractions of immune cells was assessed by the Wilcoxon test. Student’s *t*-test was used to compare the differences in the TMB and MATH scores between the high- and low-CIFI subgroups. The “ggplot2,” “ggforest,” “cowplot,” “plot3D,” “maftools,” “VennDiagram,” and “ggplotify” packages in R (version 4.0.2) were used for visualization. A *p*-value of less than 0.05 was considered to be statistically significant (^∗^*p* < 0.05; ^∗∗^*p* < 0.01; ^∗∗∗^*p* < 0.001; ^****^p < 0.0001).

## Results

### Construction of the CIFI Classifier in HCC

**FIGURE 1:**
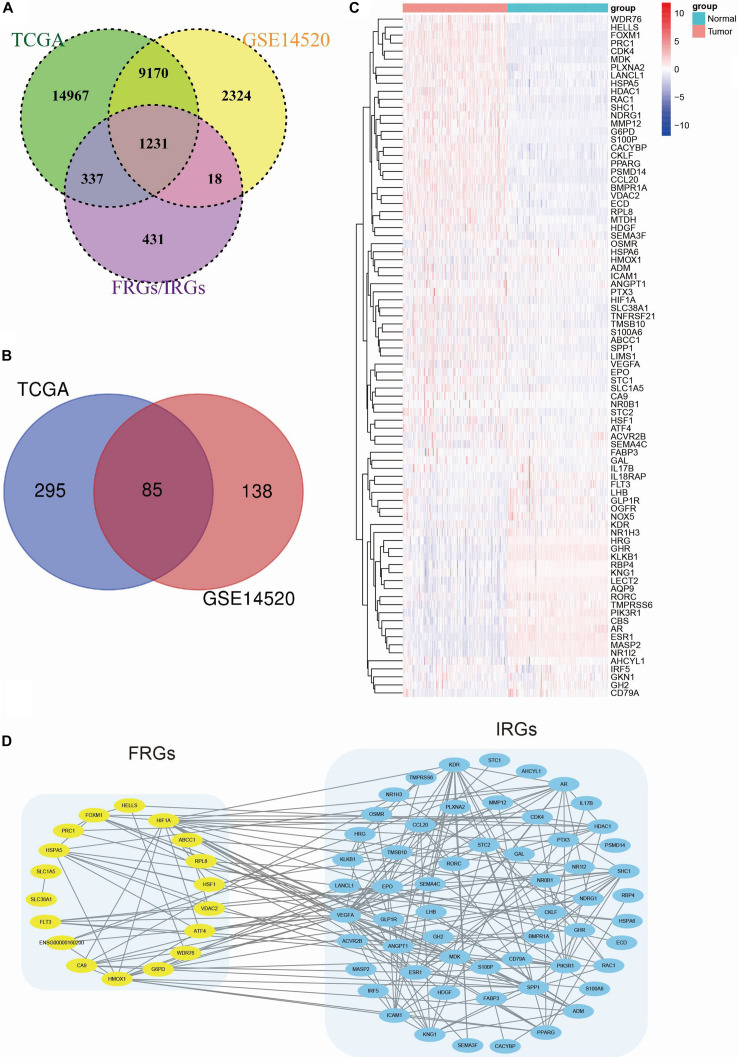
Identification of prognostic FRGs and IRGs in HCC. **(A)** A Venn diagram indicating that 1231 FRGs and IRGs were identified in the GSE14520 and TCGA cohorts. **(B)** A Venn diagram indicating that 85 prognostic genes were identified in the GSE14520 and TCGA cohorts. **(C)** A heatmap showing the expressions of the 85 prognostic genes in the tumors and normal tissues of the GSE14520 dataset. **(D)** A PPI network suggesting the relationship between FRGs and IRGs.

Populations of 220 patients from the GSE14520 dataset and 365 patients from the TCGA dataset were identified and included in this study (Table 1). The gene expression data were used to construct the CIFI classifier. In total, 279 FRGs (four genes were removed because they were identified as both DOFs and SOFs) and 952 IRGs in the two datasets were mapped and used for model construction (Figure 1A). Univariate Cox regression analysis was conducted to estimate the prognostic significance of these genes. The results showed that 85 genes in both the GSE14520 and TCGA cohorts had significant prognostic relevance (*p* < 0.05) (Figure 1B and Supplementary Table 2). The expressions of these 85 genes in the GSE14520 cohort are shown in Figure 1C. The PPI network of these genes demonstrates that the FRGs had strong correlations with the IRGs (Figure 1D).

**FIGURE 2:**
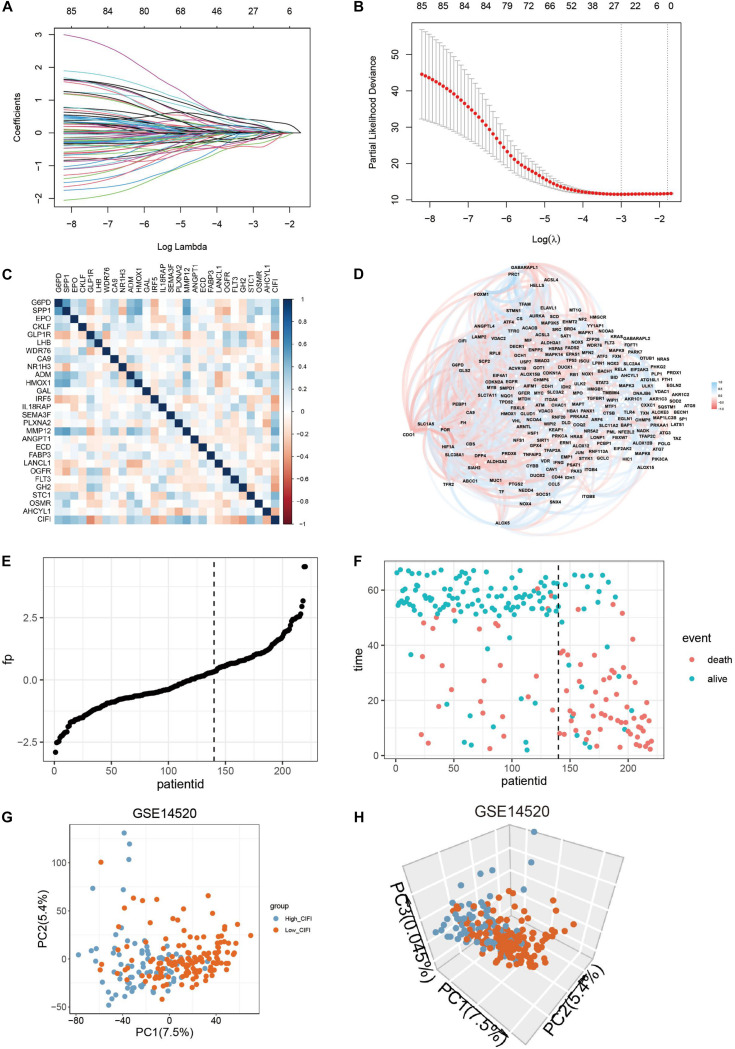
Construction of a predictive model and the CIFI of HCC. **(A,B)** The LASSO Cox regression model was constructed from the 85 prognostic genes, and the tuning parameter (λ) was calculated based on the partial likelihood deviance with 10-fold cross-validation. An optimal log λ value is indicated by the vertical black line in the plot. **(C,D)** Correlation networks **(C)** between the CIFI value and the 27 signature genes or **(D)** between the CIFI value and the ferroptosis-related genes in the GSE14520 dataset. **(E,F)** The distribution and optimal cutoff value of **(E)** the risk scores and **(F)** the OS status and OS in the GSE14520 dataset. **(G,H)** The **(G)** 2D and **(H)** 3D plots of the PCA of the GSE14520 dataset based on the expression profiles of the 27 signature genes in different risk groups.

The LASSO Cox regression model was then applied to construct a prognostic model for the OS of HCC patients by using the gene expression data of the 85 genes in the GSE14520 dataset. The model identified 27 genes based on the optimal value of λ (Figures 2A,B). Among them, G6PD, WDR76, CA9, and AHCYL1 were FRGs, HMOX1, and FLT3 participated in both ferroptosis and the immune process, and the remaining 21 genes (SPP1, EPO, CKLF, GLP1R, LHB, NR1H3, ADM, GAL, IRF5, IL18RAP, SEMA3F, PLXNA2, MMP12, ANGPT1, ECD, FABP3, LANCL1, OGFR, GH2, STC1, and OSMR) were IRGs. A novel risk score was calculated by multiplying the gene expression of each gene and its corresponding coefficient, which was obtained by multivariate Cox regression analysis. The CIFI values were generated by the formula mentioned in section “Materials and Methods.” The correlation analysis indicated that the CIFI value was significantly correlated with the selected genes (Figure 2C), and the correlations between the CIFI values and FRGs are shown in Figure 2D. The HCC patients were stratified into high-risk (high-CIFI, *n* = 80) and low-risk (low-CIFI, *n* = 140) subgroups based on the optimal CIFI cut-off value (0.52), which was calculated by the surv_cutpoint function in the “survminer” package (Figure 2E and Supplementary Figure 1A). As illustrated in Figure 2F, the patients in the high-risk subgroup had more occurrences of death and shorter survival times. PCA revealed that the patients in these two subgroups were distinctively clustered (Figures 2G,H).

**FIGURE 3:**
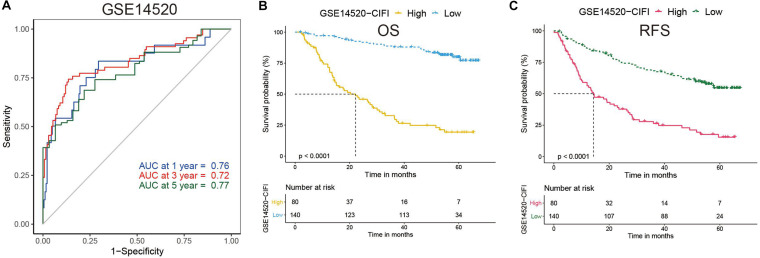
Time-dependent ROC analysis and Kaplan–Meier analysis of the CIFI-stratified patients in the GSE14520 cohort. **(A)** Time-dependent ROC analysis of the CIFI regarding the OS and survival status in the GSE14520 cohort. **(B,C)** Kaplan–Meier plots of the **(B)** OS and **(C)** RFS in the high-CIFI and low-CIFI subgroups of the GSE14520 cohort.

Time-dependent ROC curves were plotted by R software, and the AUC was calculated at different time points to estimate the predictive performance of the CIFI. As shown in Figure 3A, the AUC reached 0.76 at 1 year, 0.72 at 3 years, and 0.77 at 5 years, suggesting a favorable predictive value of the CIFI in short- and long-term follow-up. Kaplan–Meier curves indicated that patients with high CIFI values had significantly shorter OSs than their counterparts with low CIFI values (Figure 3B, *p* < 0.001). In addition, patients with high CIFI values relapsed earlier than those with low CIFI values (Figure 3C, *p* < 0.001).

### Validation of the CIFI Classifier in HCC

**FIGURE 4:**
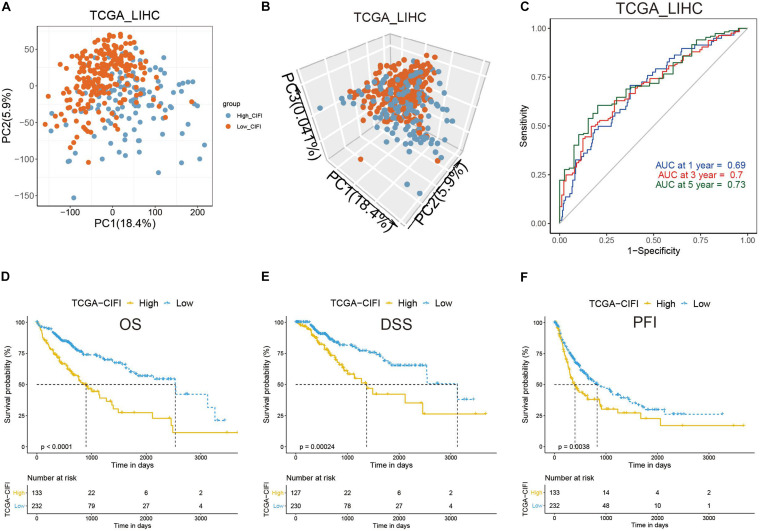
Validation of the CIFI in the TCGA cohort. **(A)** 2D and **(B)** 3D plots of the PCA of the TCGA dataset. **(C)** Time-dependent ROC analysis of the CIFI regarding the OS and survival status in the TCGA cohort. **(D–F)** Kaplan–Meier plots of the **(D)** OS, **(E)** DSS, and **(F)** PFI in the high-CIFI and low-CIFI subgroups of the TCGA cohort.

To validate the indicative CIFI value in a larger cohort of HCC patients, the CIFI values in the TCGA dataset (*n* = 365) were calculated using the same risk formula and cutoff point obtained from the GSE14520 dataset. Ultimately, 63.64% of the HCC patients (*n* = 232) in the TCGA cohort were categorized into the low-risk (low-CIFI) subgroup, while the remaining patients (*n* = 133) were categorized into the high-risk (high-CIFI) subgroup. Consistent with the results of the GSE14520 dataset, PCA indicated that the two subgroups in the TCGA cohort were distributed in discrete directions (Figures 4A,B). The AUCs for OS were 0.69 at 1 year, 0.7 at 3 years, and 0.73 at 5 years, indicating an increasing predictive value in long-term follow-up (Figure 4C). The medium OS time of the patients in the low-risk subgroup was 2,532 days, which was dramatically longer than that of patients in the high-risk subgroup (medium OS time: 899 days, *p* < 0.0001, Figure 4D). In addition, patients with low CIFIs had significantly longer DSSs (*p* = 0.00024, Figure 4E) and PFIs (*p* = 0.0038, Figure 4F).

### Ferroptosis Profile in the CIFI

**FIGURE 5:**
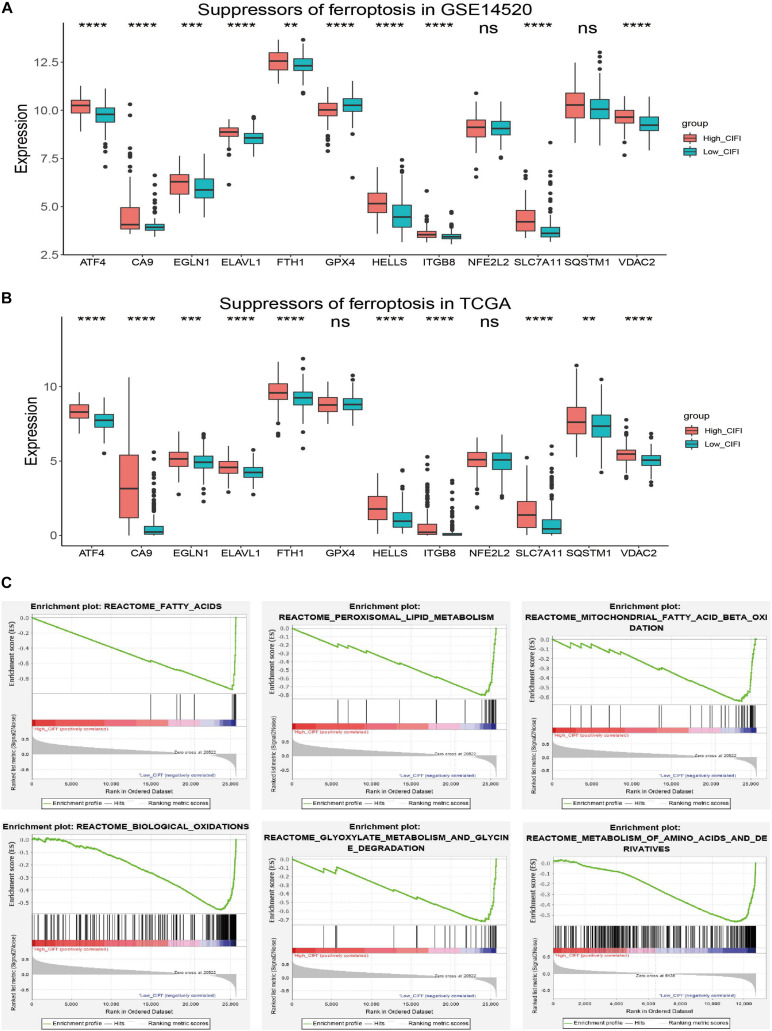
Ferroptosis profiles in the CIFI-stratified groups. **(A,B)** Comparison of the expressions of the suppressors of ferroptosis between the high- and low-CIFI subgroups of the **(A)** GSE14520 and **(B)** TCGA cohorts. **(C)** GSEA of the CIFI-stratified groups in the GSE14520 and TCGA cohorts. **p < 0.01; ***p < 0.001; ****p < 0.0001.

A correlation analysis of the GSE14520 dataset revealed a correlation between the CIFI values and FRGs (Figure 2D). Because genes might either facilitate or suppress ferroptosis, the transcriptional changes of SOFs and DOFs in the CIFI-stratified subgroups were investigated. ATF4, CA9, EGLN1, ELAVL1, FTH1, GPX4, HELLS, ITGB8, NFE2L2, SLC7A11, SQSTM1, and VDAC2 are well-investigated SOFs (Dixon et al., 2012; Sun et al., 2016b; Chen et al., 2017; Jiang et al., 2017; Zhang et al., 2018; Bai et al., 2019; Li et al., 2019). It was found that, excluding GPX4, NFE2L2, and SQSTM1, the remaining SOFs were significantly up-regulated in the high-CIFI subgroup of the GSE14520 cohort (Figure 5A). The change of these genes was further validated in the TCGA cohort (Figure 5B). Although GPX4 was found to be significantly down-regulated in the high-CIFI subgroup of the GSE14520 dataset, such a change was not observed in the TCGA cohort (Figures 5A,B). Moreover, it was found that there were no significant differences in most of the DOFs (ALOX12, ALOX12B, ALOX15, ALOXE3, BECN1, BID, GOT1, PRKAA1, and PTGS2) at the transcriptional level between the high- and low-CIFI subgroups in the GSE14520 cohort (Supplementary Figure 1B). Moreover, ALOX15B and NOX1 were significantly down-regulated in the high-CIFI subgroup, while ACSL4 and ALOX5 were significantly up-regulated (Supplementary Figure 1B).

Gene Set Enrichment Analysis was further conducted between the two subgroups of both the GSE14520 and TCGA cohorts. The results showed that the genes in the low-CIFI subgroups of the two cohorts were significantly enriched in ferroptosis-related biological processes like biological oxidation, fatty acid metabolism, peroxisome, mitochondrial fatty acid beta-oxidation, peroxisomal lipid metabolism, glyoxylate metabolism, and glycine degradation (Figure 5C). Considered together, these results suggest a ferroptosis-suppressed status of the high-CIFI subgroup.

### Immune Profile in CIFI

**FIGURE 6:**
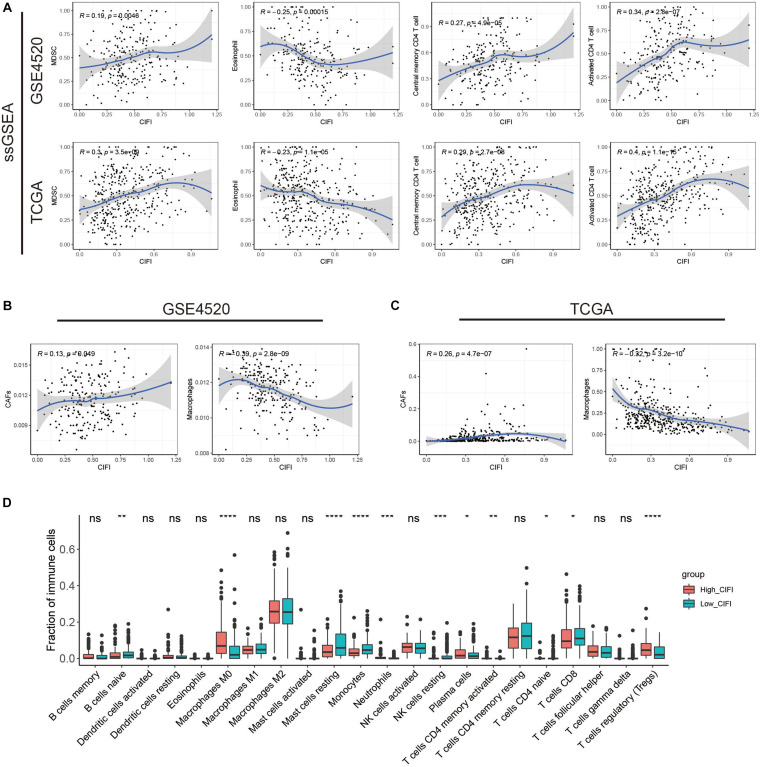
Immune profiles in the CIFI-stratified groups. **(A)** ssGSEA and correlation analysis of the CIFI value and the immune enrichment scores of immune categories in the GSE14520 and TCGA cohorts. **(B,C)** ECIP and correlation analysis of the CIFI value and the fraction of immune cells in the GSE14520 and TCGA cohorts. **(D)** Comparison between the fractions of immune cells in the high- and low-CIFI subgroups of the TCGA cohort via the CIBERSORT method. *p < 0.05; **p < 0.01; ***p < 0.001; ****p < 0.0001.

To understand how the CIFI reflects the immune status of HCC, ssGSEA was first employed to calculate the immune enrichment scores of various immune categories in each patient, and their relationships with the CIFI value were investigated. Myeloid-derived suppressor cells (MDSCs) are immune-suppressing cells that contribute to the growth and invasion of HCC, while eosinophil inhibits the growth of the disease (Lin et al., 2017; Hollande et al., 2019; Lu et al., 2019). As shown in Figure 6A, the CIFI value was found to have a significant positive correlation with MDSCs in both the GSE14520 and TCGA datasets, while a significant negative correlation was observed between the CIFI value and eosinophil in both datasets. However, the CIFI value also exhibited positive associations with activated CD4 T cells and central memory CD4 T cells (Figure 6A), which are regarded as cytotoxic cells against HCC (Jin et al., 2018). The proportion of immune cells was also estimated by the EPIC application, and it was found that the CIFI value was positively correlated with the fraction of cancer-associated fibroblasts (CAFs) and negatively correlated with the fraction of macrophages (Figures 6B,C). The GDC website^6^ contains data on the fractions of immune cells in tumors of TCGA via application of the CIBERSORT method. As shown in Figure 6D, macrophages M0, plasma cells, neutrophils, and regulatory T cells (Tregs) were up-regulated in the high-CIFI subgroup of the TCGA cohort, while naïve B cells, resting mast cells, monocytes, resting natural killer (NK) cells, naïve CD4 T cells, and CD8 T cells were significantly down-regulated (*p* < 0.05).

### Gene Mutation in CIFI

**FIGURE 7:**
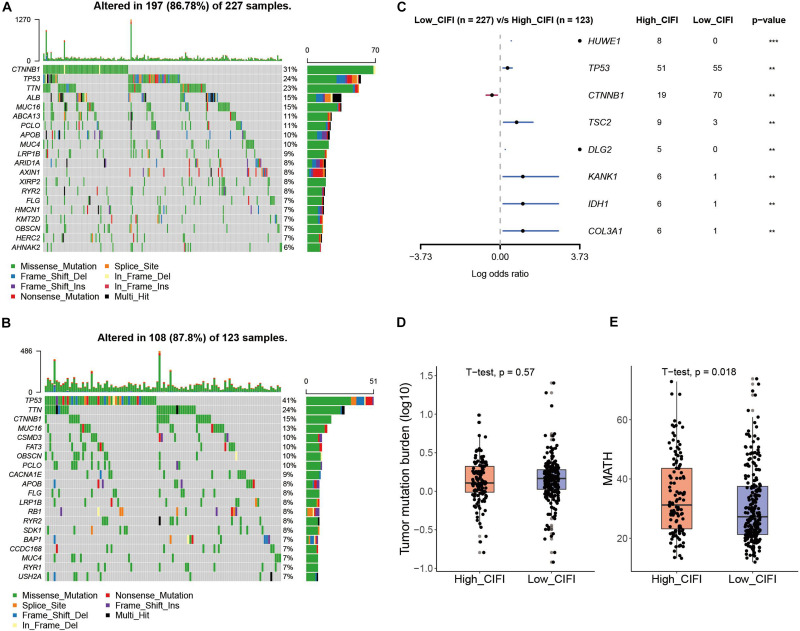
Somatic mutation in the CIFI-stratified groups. **(A,B)** Oncoplots of the mutated genes in the **(A)** low-CIFI and **(B)** high-CIFI subgroups of the TCGA cohort. **(C)** Forest plot of the differentially mutated genes between the high- and low-CIFI groups. **(D)** TMB and **(E)** MATH scores in the high- and low-CIFI groups.

To investigate the difference in gene mutation between the high- and low-CIFI subgroups, simple nucleotide variation data were downloaded from the GDC database^7^ and processed with the “maftools” package in R. Supplementary Figures 1C,D present summaries of the gene mutation information of these two subgroups. As shown in Figure 7A, the top five genes with the highest mutation frequencies in the low-CIFI subgroup were CTNNB1 (31%), TP53 (24%), TTN (23%), ALB (15%), and MUC16 (15%), while those in the high-CIFI subgroup (Figure 7B) were TP53 (41%), TTN (24%), CTNNB1 (15%), MUC16 (13%), and CSMD3 (10%). HUME1, TP53, TSC2, DLG2, KANK1, IDH1, and COL3A1 were found to be highly mutated in the high-CIFI subgroup as compared to the low-CIFI subgroup, while CTNNB1 was found to be highly mutated in the low-CIFI subgroup (Figure 7C). Although the TMBs were not different between the two subgroups (Figure 7D), high-CIFI patients had higher MATH scores, demonstrating a higher level of tumor heterogeneity in this subgroup (Figure 7E, *p* = 0.018).

### Independent Prognostic Value of the CIFI Classifier

The preceding analysis suggests that HCC patients with high CIFI values had a ferroptosis-suppressive and immune-suppressive status; this spurred an interest to analyze the associations between the CIFI value and clinicopathological features of these patients. As shown in Table 2, more patients in the high-CIFI subgroup in both the GSE14520 and TCGA cohorts were at an advanced TNM stage (stage III or IV, *p* < 0.001), and a considerably higher percentage of patients in the high-CIFI subgroup of the GSE14520 cohort were at an advanced BCLC stage (*p* = 4.88 × e^–5^). In addition, a significantly higher percentage of HCC patients in the high-CIFI subgroup of the GSE14520 cohort had cirrhosis (*p* = 0.009881), while no difference was observed in patients with fibrosis between the two subgroups of the TCGA cohort (*p* = 0.2979). Moreover, more patients in the high-CIFI subgroup of the TCGA cohort had higher histologic grades (G3 or G4, *p* < 0.001) or vascular invasion (*p* = 0.02296). High-CIFI HCC patients in the GSE14520 cohort had higher levels of AFP (*p* = 0.001172), and such a relationship was also observed in the TCGA cohort, although the result did not reach significance. No consistent differences were observed between CIFI value and age or gender in the GSE14520 and TCGA cohorts. Considered together, these results suggest that a high CIFI value is associated with worse clinicopathological features of HCC patients.

**TABLE 2:** Relationships between the CIFI value and clinicopathological features of HCC patients.

Clinicopathological features	GSE14520			TCGA		
	Low CIFI	High CIFI	*p*-Value	Low CIFI	High CIFI	*p*-Value
**Age**						
<60	107	71	0.03953	103	62	0.7637
≥60	33	9		129	71	
**Gender**						
Male	119	71	0.565	170	76	0.002301
Female	21	9		62	57	
**AFP**						
≤300	87	31	0.001172	149	63	0.05223
>300	51	48		36	28	
**Stage**						
I/II	122	48	2.49E-06	178	76	1.86E-05
III/IV	16	32		38	49	
**Cirrhosis**						
No	17	1	0.009881	–	–	–
Yes	123	79		–	–	
**BCLC**						
0/A	119	49	4.88E-05	–	–	–
B/C	19	31		–	–	
**Histologic grade**						
G1 + G2	–	–	–	162	68	0.0003118
G3 + G4	–	–		66	64	
**Vascular invasion**						
No	–	–	–	144	61	0.02296
Yes	–	–		60	46	
**Fibrosis**						
No	–	–	–	49	25	0.2979
Yes	–	–		100	35	
**Child-Pugh**						
A	–	–	–	151	65	1
B/C	–	–		15	7	

**FIGURE 8:**
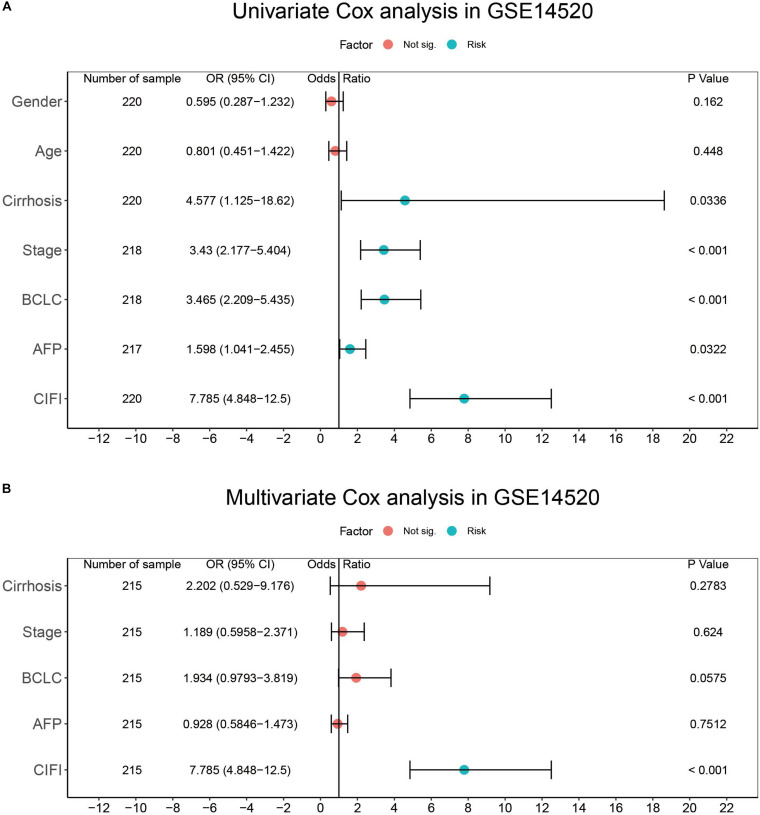
Results of the **(A)** univariate and **(B)** multivariate Cox regression analyses regarding OS in the GSE14520 cohort.

**FIGURE 9:**
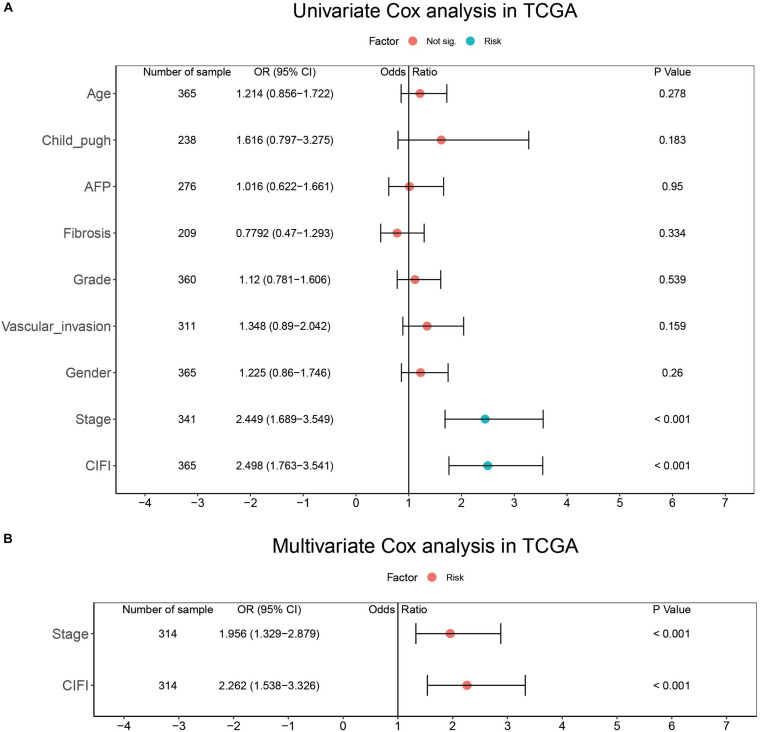
Results of the **(A)** univariate and **(B)** multivariate Cox regression analyses regarding OS in the TCGA cohort.

To determine whether the CIFI could serve as an independent prognostic predictor of OS, the clinicopathological features and CIFI values were first input into a univariate Cox regression analysis. As illustrated in Figures 8A, 9A, the CIFI value was found to be significantly associated with OS in both the GSE14520 (HR = 7.785, 95% CI = 4.848–12.5, *p* < 0.001) and TCGA datasets (HR = 2.498, 95% CI = 1.763–3.541, *p* < 0.001). Then, the CIFI values and the clinicopathological features with prognostic significance (*p* < 0.05) were subjected to a multivariate Cox regression analysis, which revealed that CIFI remained an independent prognostic predictor after correction for other confounding factors (*p* < 0.001, Figures 8B, 9B).

## Discussion

The advent of selective genome- and immune-targeted therapy has considerably improved the prognosis of cancer patients, and treatment decisions tend to be made on the basis of abnormal molecular profiles or “signatures” rather than the tissue type or anatomical site of tumors (Jackson and Chester, 2015; Abubakar and Gan, 2016; Jia et al., 2016). For instance, patients with lung cancer, colon cancer, or melanoma bearing BRAF V600E mutations all benefit from inhibitors targeting this mutation (Long et al., 2017; Planchard et al., 2017; Kopetz et al., 2019). Indeed, an increasing number of studies have attempted to identify subgroups of tumor patients based on their molecular profiles, which reflect unique phenotypes, distinct treatment responses, and different prognoses. For example, the hypoxia^low^/immune^high^ subgroup of triple-negative breast cancer (TNBC) patients has a significantly longer OS and might have a better response toward immunotherapies (Zheng et al., 2020). Colorectal cancer (CRC) patients can be classified into low- and high-risk groups on the basis of their autophagy-related features, and high-risk CRC patients might require more aggressive treatment interventions (Zhou et al., 2019).

Considering that immunotherapy and sorafenib inhibit the growth of tumors (including HCC) via the induction of ferroptosis (Louandre et al., 2013; Wang W. et al., 2019), HCC patients with distinct ferroptosis and immune phenotypes might have different prognoses. In this work, the CIFI of HCC was developed based on the currently known FRGs and IRGs and on their expression data from publicly available datasets. HCC patients with high CIFI values were found to have significantly shorter OSs, RFSs, DSSs, and PFIs than those with low CIFI values (Figures 3B,C, 4C–F). Moreover, the high-CIFI subgroup was found to have a significantly higher percentage of HCC patients with worse clinicopathological features, such as an advanced TNM stage, an advanced BCLC stage, cirrhosis, and vascular invasion (Table 2). The patients in the high-CIFI subgroup showed a ferroptosis-suppressive status, as a set of well-defined SOFs was significantly up-regulated in this subgroup (Figures 5A,B). A recent study showed that both SQSTM1 and NFE2L2 protect HCC cells from ferroptosis (Sun et al., 2016b); however, no consistent change was observed in these two genes between the high- and low-CIFI subgroups, suggesting that the SQSTM1-Keap1-NFE2L2 pathway might not play a role in the CIFI-stratified subgroups. GPX4, a key regulator in suppressing ferroptosis (Hassannia et al., 2019; Seibt et al., 2019), was unexpectedly found to be down-regulated in the high-CIFI subgroup of the GSE14520 cohort, while its expression in the TCGA cohort was not different between the high and low-CIFI subgroups. This suggests that other imbalanced factors between the two subgroups, like hepatitis B virus (HBV) or hepatitis C virus (HCV) infection, or fatty liver disease, might play a predominant role in the expression of this gene. Indeed, other studies have found that GPX4 is over-expressed in HCV-related HCC patients and could be induced by HCV to increase virion infectivity (Guerriero et al., 2015; Brault et al., 2016). However, the HCV infection status of the HCC patients in these two cohorts was not available, and further studies may be required to address this question. In addition, two typical DOFs, namely NOX1 and ALOX15B, were found to be significantly down-regulated in HCC patients with high CIFI values, while many other DOFs were not differentially expressed between the two subgroups (Supplementary Figure 1B). Interestingly, ACSL4 and ALOX5 were found to be significantly up-regulated in HCC patients with high CIFI values, even though they facilitate the execution of ferroptosis (Liu et al., 2015; Yuan et al., 2016; Doll et al., 2017). However, it is worth noting that both ACSL4 and ALOX5 are over-expressed in HCC as compared to normal livers, and promote the progression of the disease (Xu et al., 2011; Chen et al., 2020; Ndiaye et al., 2020; Wang et al., 2020). Thus, the over-expression of ACSL4 and ALOX5 contributes to the proliferation and progression of HCC, but renders the patients more susceptible to ferroptosis inducers. Indeed, researchers have found that some genes participate in multiple biological activities, some of which even seem contradictory. For instance, the well-known tumor suppressor TP53 plays an oncogenic role in HCC by inducing the P53 upregulated modulator of apoptosis (PUMA) (Kim et al., 2019). Nicotinamide phosphoribosyltransferase (NAMPT) drives tumor immune evasion by inducing PD-L1 expression in multiple types of tumors, but enhances the efficacy of immune checkpoint inhibitors (Lv et al., 2020). Further, GSEA revealed that genes in the low-CIFI subgroup were significantly enriched in biological oxidation, fatty acid metabolism, peroxisome, and glycine degradation (Figure 5C). These biological processes are all critical for the execution of ferroptosis (Hassannia et al., 2019), suggesting a high sensitivity to ferroptosis in this subgroup. In addition, recent studies have found that ferroptosis inducers, such as erastin and sorafenib, could alleviate liver fibrosis (Sui et al., 2018; Wang L. et al., 2019; Zhang Z. et al., 2020); thus, the ferroptosis-suppressive status observed in the high-CIFI subgroup might facilitate the progression of liver fibrosis and the development of cirrhosis. After all, the high-CIFI subgroup had a significantly higher percentage of HCC patients with cirrhosis (Table 2).

Hepatocellular carcinoma patients with high CIFI values also exhibited immune-suppressive features. ssGSEA revealed that the CIFI value was positively correlated with the infiltration of MDSCs, which are able to promote immune escape and impair antitumor T-cell response in HCC (Chiu et al., 2017; Lin et al., 2017). On the contrary, eosinophil, which possesses anti-tumor activity toward tumors, including HCC (Kataoka et al., 2004; Hollande et al., 2019), was found to have a significantly negative association with the CIFI value in HCC (Figure 6A). Unexpectedly, a positive association was also observed between CIFI value and CD4 T cells, which are regarded as cytotoxic to tumors (Jin et al., 2018). However, a study has shown that HBV-specific CD4 T cells are less cytotoxic to HCC cells and suppress the anti-tumor function of CD8 T cells (Meng et al., 2017). Indeed, 95.9% (211/220) of patients in the GSE14520 cohort were either active viral replication chronic carriers (AVR-CCs) or HBV chronic carriers (HBV-CCs) (Table 1). Consequently, the positive relationship between CIFI and CD4 T cells might suggest an immune-suppression status instead of an immune-activation status. CIFI was also found to have a positive relationship with the fraction of CAFs in HCC (Figure 6B). As one of the most abundant and critical components of the TME, CAFs contribute to immune evasion and immunotherapy failure, and promote the proliferation and invasion of tumors, including HCC, by secreting various growth factors and cytokines (Kubo et al., 2016; Chakravarthy et al., 2018; Liu T. et al., 2019). CIBERSORT analysis further indicated that HCC patients with high CIFI values had a significantly higher fraction of Tregs, which have been shown to be enriched in HCC and to inhibit IFN-gamma secretion and the cytotoxicity of CD8 + T cells (Yang et al., 2012; Langhans et al., 2019). Although less lytic than activated NK cells, resting NK cells are still cytotoxic and target cells like tumor cells, and could be converted to activated NK cells with certain stimuli (Bryceson et al., 2006; Lugini et al., 2012); however, the CIBERSORT analysis also revealed that the high-CIFI subgroup exhibited a lower infiltration of resting NK cells, while no difference was observed in the activated NK cells between the two subgroups (Figure 6D). In addition, although the TMB was not found to be different between the two subgroups based on the calculation of gene mutation data, MATH analysis suggested that the high-CIFI subgroup had higher levels of tumor heterogeneity, which is generally a predictor of worse prognosis and is correlated with less immune response in various types of cancer (Mroz et al., 2015; Ma et al., 2019; McDonald et al., 2019). Considered together, these data suggest that high CIFI values might be correlated with immunosuppression in HCC.

In particular, it was observed that patients in the high-CIFI subgroup had a significantly higher frequency of TP53 mutation (41 vs. 24%). TP53 is a typical tumor suppressor, and its mutation leads to tumorigenesis and the progression of many types of tumors, including HCC (Leroy et al., 2014). However, TP53 mutation could either induce or suppress ferroptosis depending on the mutation site of the gene (Jiang et al., 2015; Jennis et al., 2016; Ou et al., 2016), and further studies are required to illustrate its exact impact on ferroptosis in HCC. Moreover, TP53 can activate an anti-tumor immune response via multiple mechanisms, like the down-regulation of immune-evading signals such as PD-L1, or the up-regulation of the NK cell ligands ULBP1 and ULBP2 (Textor et al., 2011; Cortez et al., 2016; Munoz-Fontela et al., 2016). HCC patients with TP53^WT^ have a significantly stronger local immuno-phenotype than those with mutant TP53 (Long et al., 2019). Consequently, the higher frequency of TP53 mutation observed in the HCC patients in the high-CIFI subgroup might contribute to the ferroptosis- and immune-suppressive phenotype of the subgroup.

A recent study predicted the prognosis of HCC patients by constructing a FRG signature from 60 FRGs (Liang et al., 2020). However, the results were not repeated and the signature was not compared with the model developed in the present work because the CARS gene could not be found in TCGA, GSE14520, or the Gene Cards website.^8^ The authors might have mistaken CARS1 or CARS2 for CARS. In addition, the AUCs of the 10-gene signature in the TCGA and ICGC cohorts were respectively 0.668 and 0.718 at 3 years; in contrast, those determined by the model in the present work were 0.72 and 0.7 at 3 years in the GSE14520 and TCGA cohorts, suggesting a slightly better predictability of the proposed model. In addition, the proposed signature was also found to have good predictability in long-term follow-up, as it achieved AUCs of 0.77 and 0.73 at 5 years in the GSE14520 and TCGA cohorts, respectively. Moreover, not only IRGs, but also FRGs (*n* = 283), were included in this work, and both suppressors and DOFs were considered for the construction of the predictive model. Therefore, the model might more comprehensively reflect the ferroptosis and immune status of HCC patients.

The predictability of the proposed signature failed to be validated in the ICGC cohort because only a few dozen of the samples included the gene expression data of the selected genes. On the other hand, it should be noted that this work was limited because it was a retrospective study; thus, a further well-designed prospective analysis is necessary to validate the value of the developed model.

Because immunotherapy and targeted therapies, like sorafenib, function through ferroptosis, the novel comprehensive ferroptosis–immune status classifier developed in the present study suggests that personalized treatment should be applied in different subgroups of HCC patients. The high-CIFI subgroup represents a ferroptosis- and immune-suppressive phenotype, and might not benefit greatly from immunotherapy, targeted therapy, or a combined therapy like atezolizumab plus bevacizumab. On the other hand, the distinct features of this subgroup also imply that a ferroptosis inhibitor might synergize with immunotherapy and targeted therapy for a better treatment response.

## Conclusion

In conclusion, a novel prognostic classifier based on ferroptosis and immune expression profiles in HCC was developed and validated. This classifier could be used for prognostic prediction and the selection of patients for immunotherapies and targeted therapies.

## Data Availability Statement

The original contributions presented in the study are included in the article/Supplementary Material, further inquiries can be directed to the corresponding author/s.

## Author Contributions

ZS conceived and designed the study and drafted the manuscript. YL, XZ, and ZS carried out the data analysis. ZS and JL conducted the bioinformatics analysis with R software. YL and JT conducted the searching and reading of the existing literature. All authors revised the article and approved the final version to be published.

## Conflict of Interest

The authors declare that the research was conducted in the absence of any commercial or financial relationships that could be construed as a potential conflict of interest.

## Footnotes

1 http://www.zhounan.org/ferrdb/

2 https://pubmed.ncbi.nlm.nih.gov/

3 https://www.immport.org

4 http://string-db.org

5 https://gfellerlab.shinyapps.io/EPIC_1-1/

6 https://gdc.cancer.gov/about-data/publications/panimmune

7 https://portal.gdc.cancer.gov

8 https://www.genecards.org/
