# Supplementary material for: Development and Validation of a Combined Ferroptosis and Immune Prognostic Classifier for Hepatocellular Carcinoma
Source: Front Cell Dev Biol. 2020 Dec 23;8:596679. doi: 10.3389/fcell.2020.596679 (PMC7785857; doi:10.3389/fcell.2020.596679)

Supplementary tables

Supplementary Table 1. Summary of ferroptosis-related genes.

| Ferroptosis-related genes | | | |
| --- | --- | --- | --- |
| Drivers of ferroptosis | | Suppressors of ferroptosis | |
| Symbol | PMID | Symbol | PMID |
| ABHD12 | 32195565 | ABCC1 | 30726737 |
| POLG | 32186434 | ACACB | 32029897 |
| ACSL3 | 30686757 | ACO1 | 27514700 |
| AIFM2 | 31634899 | ACSF2 | 22632970 |
| AKR1C1 | 31780644 | ACSL4 | 25965523 |
| AKR1C2 | 24844246 | ACVR1B | 30804470 |
| AKR1C3 | 31780644 | AHCYL1 | 32144268 |
| ALDH3A1 | 30333913 | AIFM1 | 29378335 |
| ALDH3A2 | 32458004 | ALOX12 | 27506793 |
| ARF6 | 32368394 | ALOX12B | 27506793 |
| ARNTL | 31355331 | ALOX15 | 27506793 |
| ATF4 | 28553953 | ALOX15B | 27506793 |
| ATRA | 28900510 | ALOX15B | 27506793 |
| AURKA | 31740746 | ALOX5 | 26235588 |
| BRD4 | 30988278 | ALOXE3 | 27506793 |
| BRD4 | 30988278 | ALOXE3 | 27506793 |
| CA9 | 31442913 | ANGPTL4 | 31641008 |
| CAV1 | 31877357 | ANO6 | 31060306 |
| CBS | 31819185 | ARF | 28985506 |
| CCL5 | 32567935 | ATF3 | 31273299 |
| CD44 | 30709928 | ATG13 | 27514700 |
| CDH1 | 31341276 | ATG16L1 | 27514700 |
| CDKN1A | 29346757 | ATG16L1 | 31679460 |
| CDKN1A | 29346757 | ATG3 | 27514700 |
| CHMP5 | 31761326 | ATG4D | 27514700 |
| CHMP6 | 31761326 | ATG5 | 27245739 |
| CISD1 | 27510639 | ATG7 | 27245739 |
| CISD2 | 29928961 | ATM | 31320750 |
| COQ2 | 31634900 | ATP5MC3 | 22632970 |
| CP | 32283255 | BACH1 | 31740582 |
| D2HGDH | 31591388 | BAP1 | 30202049 |
| DECR1 | 32427840 | BECN1 | 27514700 |
| EGLN1 | 28900510 | BID | 28384611 |
| EHMT2 | 30988278 | CARS1 | 26184909 |
| EIF2AK3 | 31519193 | CDKN2A | 28985506 |
| EIF4A1 | 32190008 | CDO1 | 29144989 |
| ENPP2 | 29551679 | CGAS | 32186434 |
| FADS2 | 28900510 | CHAC1 | 29383104 |
| FANCD2 | 27773819 | CS | 22632970 |
| FBXL5 | 22632970 | CTSB | 30031610 |
| FH | 29917289 | CXXC1 | 29588351 |
| FOXM1 | 31974380 | CYBB | 22632970 |
| FTH1 | 30557609 | DLD | 31931284 |
| FTMT | 28066232 | DNAJB6 | 31701262 |
| FXN | 32169822 | DPP4 | 28813679 |
| GABPB1 | 31700067 | DUOX1 | 22632970 |
| GCH1 | 31989025 | DUOX2 | 22632970 |
| GCLC | 26166707 | EGFR | 28297659 |
| GDF15 | 32209255 | EGLN2 | 31355331 |
| GFER | 30993878 | EIF2AK2 | 30817950 |
| GLUD1 | 28900510 | ELAVL1 | 30081711 |
| GPX4 | 31685805 | EMC2 | 22632970 |
| HELLS | 28900510 | EMP1 | 31484063 |
| HIF1A | 31355331 | EPAS1 | 30962421 |
| HMOX1 | 28515173 | ERN1 | 32593899 |
| HNF4A | 31108460 | FBXW7 | 31679460 |
| HNRNPA1 | 32106859 | FDFT1 | 27159577 |
| HSF1 | 25728673 | FLT3 | 24739485 |
| HSPA5 | 31519193 | G6PD | 22632970 |
| HSPB1 | 25728673 | GABARAPL1 | 27514700 |
| HSPB1 | 25728673 | GABARAPL2 | 27514700 |
| IDH2 | 32089268 | GLS2 | 26166707 |
| ISCU | 30557609 | GOT1 | 26166707 |
| ITGA6 | 28972104 | HBA1 | 31108460 |
| ITGB4 | 28972104 | HDDC3 | 31877357 |
| ITGB8 | 32196629 | HIC1 | 31108460 |
| JUN | 31394193 | HIF1A | 30962421 |
| KDM3B | 32107878 | HILPDA | 30962421 |
| LAMP2 | 31672277 | HMGB1 | 31105999 |
| LATS1 | 31341276 | HMGCR | 27159577 |
| LATS2 | 31341276 | HMOX1 | 26405158 |
| MAPT | 28886009 | HRAS | 26157704 |
| MIF | 29769287 | HSC70 | 30718432 |
| MT1G | 27015352 | HSP90 | 30718432 |
| MTF1 | 31320750 | IDH1 | 31591388 |
| MTOR | 29127238 | IDO1 | 31945497 |
| MUC1 | 26930718 | IFNG | 31043744 |
| MYC | 28900510 | IREB2 | 22632970 |
| NADK | 32462112 | KEAP1 | 28805788 |
| NCOA3 | 32536370 | KRAS | 26157704 |
| NEDD4 | 31974380 | LONP1 | 31822343 |
| NF2 | 31341276 | LPCAT3 | 25965523 |
| NFE2L2 | 26403645 | LPIN1 | 31061954 |
| NFS1 | 29168506 | MAP1LC3A | 27514700 |
| NQO1 | 26403645 | MAP1LC3B | 31659150 |
| NR5A2 | 32536370 | MAP3K5 | 28887319 |
| OTUB1 | 30709928 | MAPK1 | 29330409 |
| PANX2 | 32547072 | MAPK14 | 29436589 |
| PARK7 | 32144268 | MAPK3 | 29330409 |
| PAX3 | 31926626 | MAPK8 | 29330409 |
| PCBP1 | 32438524 | MAPK9 | 29330409 |
| PLIN2 | 31520166 | MDM2 | 32079652 |
| PLP1 | 31585094 | MDMX | 32079652 |
| PML | 29081404 | MFN2 | 32593899 |
| PRDX1 | 31901729 | MIOX | 31437128 |
| PRDX5 | 31700067 | MPO | 32327603 |
| PRDX6 | 31036877 | MTDH | 31527591 |
| PROM2 | 31735663 | MYB | 29144989 |
| PSAT1 | 31108460 | NAA38 | 30726737 |
| RB1 | 25444922 | NCOA4 | 27514700 |
| RELA | 32015337 | NOX1 | 22632970 |
| RNF113A | 32152280 | NOX2 | 31641008 |
| RNF20 | 31267712 | NOX3 | 22632970 |
| SAHH | 32144268 | NOX4 | 31196626 |
| SCD | 31270077 | NOX5 | 22632970 |
| SESN2 | 31323261 | NRAS | 26157704 |
| SIAH2 | 32042051 | PANX1 | 31694915 |
| SLC2A14 | 28900510 | PEBP1 | 29053969 |
| SLC2A4 | 28900510 | PGD | 22632970 |
| SLC3A2 | 26945935 | PHKG2 | 27506793 |
| SLC40A1 | 27441659 | PHKG2 | 27506793 |
| SLC7A11 | 29274359 | PIK3CA | 24739485 |
| SOD2 | 28900510 | POR | 32080622 |
| SP1 | 31056284 | PRC1 | 30907299 |
| SQSTM1 | 30339884 | PRKAA1 | 30057310 |
| SRC | 28972104 | PRKAA2 | 30057310 |
| STAT3 | 30811078 | PRKAA2 | 30057310 |
| STMN1 | 31108460 | PRKCA | 27189756 |
| STYK1 | 31542233 | PTGS2 | 31533781 |
| TFAM | 32186434 | RAB7A | 30545638 |
| TFAP2A | 32432738 | RPL8 | 22632970 |
| TFAP2C | 31056284 | SAT1 | 27698118 |
| TMBIM4 | 31507082 | SCP2 | 25402683 |
| TP53 | 29346757 | SIRT1 | 31610175 |
| TP63 | 29212036 | SLC11A2 | 32535745 |
| TPD52 | 30545638 | SLC1A5 | 26166707 |
| TXN | 30709928 | SLC38A1 | 26166707 |
| VDAC2 | 29569437 | SMAD3 | 32471991 |
| VDR | 31996668 | SMPD1 | 29282302 |
| VHL | 29872221 | SNX4 | 27514700 |
| WDR76 | 28900510 | SOCS1 | 29081404 |
| ZFP36 | 31679460 | STING1 | 32186434 |
|  |  | TAZ | 31641008 |
|  |  | TF | 26166707 |
|  |  | TFR2 | 26166707 |
|  |  | TFRC | 27514700 |
|  |  | TGFBR1 | 30804470 |
|  |  | TLR4 | 31196626 |
|  |  | TNFAIP3 | 31160087 |
|  |  | TP53 | 30321484 |
|  |  | ULK1 | 27514700 |
|  |  | ULK2 | 27514700 |
|  |  | USP7 | 31267712 |
|  |  | VDAC1 | 30421242 |
|  |  | VDAC2 | 22632970 |
|  |  | VDAC3 | 31974380 |
|  |  | WIPI1 | 27514700 |
|  |  | WIPI2 | 27514700 |
|  |  | YWHAE | 31581313 |
|  |  | YY1AP1 | 31341276 |
|  |  | ZEB1 | 28678785 |

Supplementary Table 2. Eighty-five genes with significant prognostic relevance in the GSE14520 and TCGA datasets.

| Symbol | GSE14520 | | TCGA | |
| --- | --- | --- | --- | --- |
|  | HR (95% CI for HR) | *p*-value | HR (95% CI for HR) | *p*-value |
| ABCC1 | 1.3 (1.1-1.6) | 0.01 | 1.3 (1.1-1.5) | 0.00035 |
| ACVR2B | 1.6 (1.1-2.4) | 0.017 | 1.6 (1.2-2.1) | 0.0023 |
| ADM | 1.4 (1.2-1.6) | 4.40E-05 | 1.2 (1.1-1.4) | 0.0021 |
| AHCYL1 | 0.69 (0.51-0.94) | 0.017 | 1.3 (1-1.7) | 0.048 |
| ANGPT1 | 2 (1.4-2.9) | 0.00033 | 1.4 (1.1-1.7) | 0.013 |
| AQP9 | 0.84 (0.77-0.92) | 0.00022 | 0.92 (0.87-0.98) | 0.013 |
| AR | 0.8 (0.64-0.99) | 0.037 | 0.88 (0.79-0.98) | 0.022 |
| ATF4 | 1.6 (1.1-2.3) | 0.01 | 1.5 (1.1-1.9) | 0.0026 |
| BMPR1A | 1.6 (1.2-2.1) | 0.0024 | 1.5 (1.1-2) | 0.0077 |
| CA9 | 1.4 (1.2-1.7) | 1.10E-05 | 1.1 (1-1.2) | 0.00088 |
| CACYBP | 1.4 (1-1.9) | 0.03 | 1.6 (1.3-2) | 2.90E-05 |
| CBS | 0.82 (0.71-0.95) | 0.0079 | 0.77 (0.64-0.93) | 0.0076 |
| CCL20 | 1.1 (1-1.3) | 0.029 | 1.1 (1-1.2) | 0.0061 |
| CD79A | 0.45 (0.25-0.81) | 0.0079 | 0.88 (0.77-1) | 0.047 |
| CDK4 | 1.5 (1.1-2) | 0.0089 | 1.5 (1.2-1.8) | 1.50E-05 |
| CKLF | 1.3 (1-1.6) | 0.03 | 1.5 (1.3-1.9) | 3.20E-05 |
| ECD | 1.6 (1.1-2.4) | 0.02 | 1.5 (1.1-2) | 0.015 |
| EPO | 2.1 (1.6-2.7) | 7.30E-08 | 1.2 (1.1-1.3) | 3.30E-06 |
| ESR1 | 0.73 (0.57-0.93) | 0.012 | 0.74 (0.59-0.92) | 0.0078 |
| FABP3 | 1.2 (1-1.5) | 0.042 | 1.1 (1-1.2) | 0.016 |
| FLT3 | 0.24 (0.086-0.7) | 0.0085 | 0.53 (0.3-0.94) | 0.029 |
| FOXM1 | 1.4 (1-1.8) | 0.035 | 1.3 (1.1-1.5) | 3.00E-05 |
| G6PD | 1.3 (1.1-1.6) | 0.00027 | 1.4 (1.2-1.5) | 1.30E-09 |
| GAL | 1.8 (1.2-2.7) | 0.0072 | 1.3 (1.1-1.6) | 0.0027 |
| GH2 | 0.2 (0.064-0.64) | 0.0063 | 1.7 (1.1-2.8) | 0.029 |
| GHR | 0.88 (0.79-0.99) | 0.033 | 0.83 (0.74-0.93) | 0.0019 |
| GKN1 | 0.21 (0.067-0.69) | 0.0099 | 69 (5.6-860) | 0.00094 |
| GLP1R | 0.34 (0.18-0.63) | 0.00077 | 1.4 (1.2-1.7) | 5.00E-04 |
| HDAC1 | 1.5 (1.1-2.1) | 0.02 | 1.8 (1.4-2.3) | 2.60E-06 |
| HDGF | 1.9 (1.1-3.3) | 0.016 | 2 (1.5-2.6) | 8.10E-06 |
| HELLS | 1.3 (1-1.6) | 0.026 | 1.4 (1.2-1.6) | 0.00045 |
| HIF1A | 1.4 (1.1-1.8) | 0.0082 | 1.2 (1.1-1.4) | 0.0067 |
| HMOX1 | 1.3 (1.1-1.6) | 0.0013 | 1.2 (1.1-1.4) | 0.0025 |
| HRG | 0.9 (0.82-0.99) | 0.023 | 0.93 (0.88-0.98) | 0.0064 |
| HSF1 | 1.6 (1.1-2.2) | 0.01 | 1.4 (1.1-1.8) | 0.0015 |
| HSPA5 | 1.7 (1.1-2.6) | 0.017 | 1.3 (1-1.6) | 0.032 |
| HSPA6 | 1.3 (1.1-1.7) | 0.012 | 1.2 (1.1-1.3) | 0.0026 |
| ICAM1 | 1.4 (1.1-1.6) | 0.0012 | 1.1 (1-1.3) | 0.027 |
| IL17B | 1.8 (1.1-3.1) | 0.029 | 2.2 (1.2-3.8) | 0.009 |
| IL18RAP | 0.38 (0.17-0.88) | 0.024 | 0.56 (0.38-0.83) | 0.004 |
| IRF5 | 0.36 (0.2-0.66) | 0.00087 | 1.5 (1.1-1.9) | 0.003 |
| KDR | 0.63 (0.45-0.87) | 0.0046 | 0.85 (0.73-0.99) | 0.04 |
| KLKB1 | 0.8 (0.69-0.93) | 0.004 | 0.85 (0.75-0.97) | 0.012 |
| KNG1 | 0.87 (0.78-0.98) | 0.022 | 0.9 (0.84-0.97) | 0.0049 |
| LANCL1 | 0.64 (0.45-0.9) | 0.011 | 1.3 (1-1.6) | 0.02 |
| LECT2 | 0.9 (0.82-0.98) | 0.012 | 0.89 (0.83-0.95) | 0.00048 |
| LHB | 0.4 (0.18-0.89) | 0.025 | 1.6 (1.2-2) | 0.00058 |
| LIMS1 | 1.7 (1.2-2.4) | 0.0015 | 1.4 (1.1-1.7) | 0.0019 |
| MASP2 | 0.8 (0.69-0.92) | 0.0027 | 0.89 (0.83-0.97) | 0.0042 |
| MDK | 1.2 (1-1.4) | 0.025 | 1.1 (1-1.2) | 0.028 |
| MMP12 | 1.2 (1.1-1.4) | 8.50E-05 | 1.1 (1-1.3) | 0.009 |
| MTDH | 1.4 (1.1-1.9) | 0.017 | 1.3 (1-1.6) | 0.038 |
| NDRG1 | 1.4 (1.2-1.6) | 0.00014 | 1.3 (1.1-1.5) | 6.60E-05 |
| NOX5 | 0.42 (0.18-0.96) | 0.039 | 5.8 (1.2-28) | 0.03 |
| NR0B1 | 1.3 (1-1.6) | 0.042 | 1.4 (1.2-1.5) | 9.60E-07 |
| NR1H3 | 0.73 (0.55-0.97) | 0.027 | 1.7 (1.2-2.3) | 0.0013 |
| NR1I2 | 0.8 (0.7-0.92) | 0.0017 | 0.88 (0.8-0.98) | 0.014 |
| OGFR | 0.53 (0.31-0.9) | 0.019 | 1.4 (1-1.9) | 0.025 |
| OSMR | 2 (1.2-3.1) | 0.0053 | 1.1 (1-1.3) | 0.042 |
| PIK3R1 | 0.78 (0.64-0.95) | 0.012 | 0.85 (0.72-0.99) | 0.04 |
| PLXNA2 | 1.6 (1.2-2.1) | 0.0016 | 1.4 (1.1-1.8) | 0.0086 |
| PPARG | 1.3 (1-1.6) | 0.019 | 1.3 (1.1-1.5) | 0.002 |
| PRC1 | 1.3 (1-1.6) | 0.017 | 1.3 (1.1-1.5) | 0.00023 |
| PSMD14 | 1.5 (1.1-2.2) | 0.019 | 1.9 (1.5-2.5) | 8.20E-07 |
| PTX3 | 1.5 (1-2.2) | 0.045 | 1.2 (1-1.4) | 0.026 |
| RAC1 | 2 (1.2-3.3) | 0.01 | 1.8 (1.4-2.3) | 8.20E-06 |
| RBP4 | 0.81 (0.66-0.98) | 0.035 | 0.87 (0.8-0.95) | 0.0017 |
| RORC | 0.61 (0.42-0.88) | 0.0074 | 0.84 (0.75-0.94) | 0.002 |
| RPL8 | 1.4 (1-2) | 0.027 | 1.2 (1-1.4) | 0.011 |
| S100A6 | 1.2 (1.1-1.4) | 0.0027 | 1.1 (1-1.2) | 0.013 |
| S100P | 1.1 (1-1.2) | 0.015 | 1.1 (1-1.1) | 0.021 |
| SEMA3F | 1.5 (1.1-2.1) | 0.0099 | 1.4 (1.1-1.9) | 0.0056 |
| SEMA4C | 1.8 (1.2-2.7) | 0.0051 | 1.2 (1-1.5) | 0.048 |
| SHC1 | 1.4 (1-1.8) | 0.029 | 1.5 (1.2-1.8) | 0.00077 |
| SLC1A5 | 1.7 (1.1-2.5) | 0.019 | 1.3 (1.2-1.5) | 1.30E-07 |
| SLC38A1 | 1.3 (1.1-1.5) | 0.0016 | 1.3 (1.1-1.4) | 3.60E-05 |
| SPP1 | 1.2 (1.1-1.3) | 0.00079 | 1.1 (1.1-1.2) | 3.20E-06 |
| STC1 | 1.5 (1.2-1.8) | 4.00E-05 | 1.2 (1-1.3) | 0.031 |
| STC2 | 1.9 (1.2-2.9) | 0.0067 | 1.4 (1.2-1.5) | 3.90E-06 |
| TMPRSS6 | 0.79 (0.64-0.97) | 0.026 | 0.87 (0.8-0.95) | 0.0025 |
| TMSB10 | 1.4 (1.1-1.8) | 0.005 | 1.2 (1-1.3) | 0.011 |
| TNFRSF21 | 1.2 (1-1.4) | 0.017 | 1.2 (1-1.3) | 0.0059 |
| VDAC2 | 1.7 (1.2-2.5) | 0.006 | 1.6 (1.2-2.2) | 5.00E-04 |
| VEGFA | 1.5 (1.2-2) | 0.0013 | 1.3 (1.1-1.6) | 0.0025 |
| WDR76 | 1.9 (1.1-3.1) | 0.018 | 1.3 (1.1-1.5) | 0.00074 |

Supplementary figure captions

Supplementary Figure 1. (A) The optimal cutoff value of the CIFI determined by the surv_cutpoint function in the “survminer” package. (B) The expressions of drivers of ferroptosis in the high- and low-CIFI groups in the GSE14520 dataset. (C-D) Summary of gene mutation information in the (C) high-CIFI group and (D) low-CIFI group in the TCGA dataset.

Supplementary Figure 1


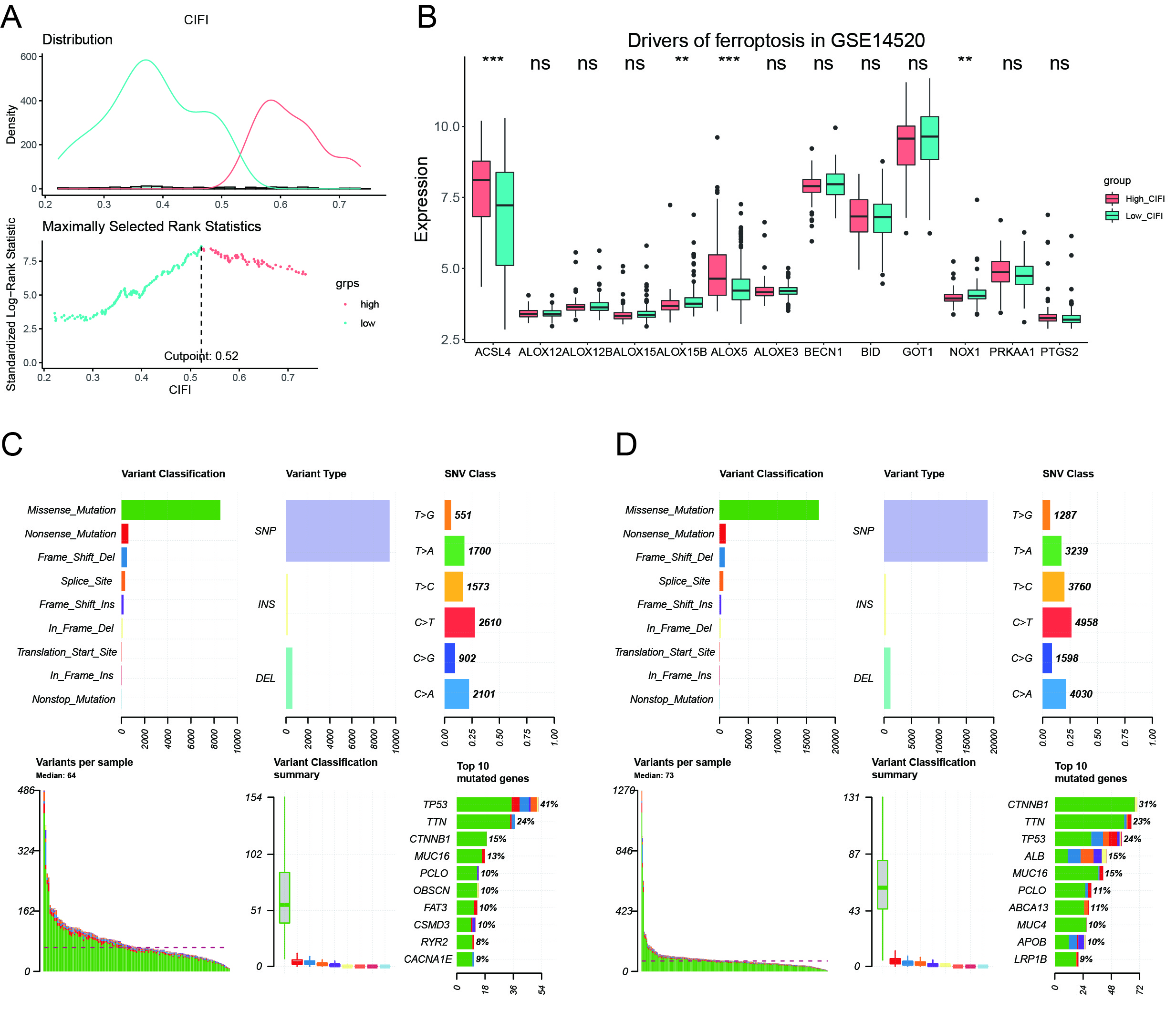

Supplement: Supplementary file 1 [file Data_Sheet_1.docx]
